# Diabetes Care, Glycemic Control, Complications, and Concomitant Autoimmune Diseases in Children with Type 1 Diabetes in Turkey: A Multicenter Study

**DOI:** 10.4274/Jcrpe.893

**Published:** 2013-03-21

**Authors:** Damla Gökşen Şimşek, Zehra Aycan, Samim Özen, Semra Çetinkaya, Cengiz Kara, Saygın Abalı, Korcan Demir, Özgül Tunç, Ahmet Uçaktürk, Gülgün Asar, Firdevs Baş, Ergun Çetinkaya, Murat Aydın, Gülay Karagüzel, Zerrin Orbak, Zerrin Orbak, Zeynep Şıklar, Ayça Altıncık, Ayşenur Ökten, Behzat Özkan, Gönül Öçal, Serap Semiz, İlknur Arslanoğlu, Olcay Evliyaoğlu, Rüveyde Bundak, Şükran Darcan

**Affiliations:** 1 Ege University School of Medicine, Department of Pediatric Endocrinology, İzmir, Turkey; 2 Dr.Sami Ulus Training and Research Hospital, Department of Pediatric Endocrinology, Ankara, Turkey; 3 Ondokuz Mayıs University School of Medicine, Department of Pediatric Endocrinology, Samsun, Turkey; 4 Istanbul University School of Medicine, Department of Pediatrics, İstanbul, Turkey; 5 Dokuz Eylül University School of Medicine, Department of Pediatric Endocrinology, İzmir, Turkey; 6 Dışkapı Pediatric Training and Research Hospital, Department of Pediatric Endocrinology, Ankara, Turkey; 7 İstanbul University School of Medicine, Department of Pediatric Endocrinology, İstanbul, Turkey; 8 Karadeniz Technical University School of Medicine, Department of Pediatric Endocrinology, Trabzon, Turkey; 9 Atatürk University School of Medicine, Department of Pediatric Endocrinology, Erzurum, Turkey; 10 Ankara University School of Medicine, Department of Pediatric Endocrinology, Ankara, Turkey; 11 Pamukkale University School of Medicine, Department of Pediatric Endocrinology, Denizli, Turkey; 12 Düzce University School of Medicine, Department of Pediatric Endocrinology, Düzce, Turkey; 13 Kırıkkale University School of Medicine, Department of Pediatric Endocrinology, Kırıkkale, Turkey

**Keywords:** type 1 diabetes, children, complications, Turkey

## Abstract

**Objective:** Epidemiologic and clinical features of type 1 diabetes mellitus (T1DM) may show substantial differences among countries. The primary goal in the management of T1DM is to prevent micro- and macrovascular complications by achieving good glycemic control. The present study aimed to assess metabolic control, presence of concomitant autoimmune diseases, and of acute and long-term complications in patients diagnosed with T1DM during childhood and adolescence. The study also aimed to be a first step in the development of a national registry system for T1DM, in Turkey.

**Methods:** Based on hospital records, this cross-sectional, multicenter study included 1 032 patients with T1DM from 12 different centers in Turkey, in whom the diagnosis was established during childhood. Epidemiological and clinical characteristics of the patients were recorded. Metabolic control, diabetes care, complications, and concomitant autoimmune diseases were evaluated.

**Results:** Mean age, diabetes duration, and hemoglobin A1c level were 12.5±4.1 years, 4.7±3.2 years, and 8.5±1.6%, respectively. Acute complications noted in the past year included ketoacidosis in 5.2% of the patients and severe hypoglycemia in 4.9%. Chronic lymphocytic thyroiditis was noted in 12%, Graves’ disease in 0.1%, and celiac disease in 4.3% of the patients. Chronic complications including neuropathy, retinopathy, and persistent microalbuminuria were present in 2.6%, 1.4%, and 5.4% of the patients, respectively. Diabetic nephropathy was not present in any of the patients. Mean diabetes duration and age of patients with neuropathy, retinopathy and microalbuminuria were significantly different from the patients without these long-term complications (p<0.01). A significant difference was found between pubertal and prepubertal children in terms of persistent microalbuminuria and neuropathy (p=0.02 and p<0.001, respectively). Of the patients, 4.4% (n:38) were obese and 5% had short stature; 17.4% of the patients had dyslipidemia, and 14% of the dyslipidemic patients were obese.

**Conclusions:** Although the majority of the patients in the present study were using insulin analogues, poor glycemic control was common, and chronic complications were encountered.

**Conflict of interest:**None declared.

## INTRODUCTION

Absence of adequate retrospective data and lack of record keeping in health care systems have been identified as important problems. Registry systems for follow-up of patients with type 1 diabetes mellitus (T1DM) have been in practice in many countries for years. With these systems, it has been possible to monitor quality of life in diabetic patients nationally, to obtain data about metabolic control and clinical course in these patients, and to develop standards for the treatment and monitoring of diabetes (1).

With this cross-sectional, multicenter study, we aimed to assess metabolic control, presence of concomitant autoimmune diseases as well as the microvascular and acute complications of diabetes in patients diagnosed with T1DM during childhood and adolescence. This assessment was considered to be a first step in the development of a registry system, as well as an investigation on the state of T1DM in Turkey.

## METHODS

The diagnosis of T1DM was based on hyperglycemia (>200 mg/dL) and related symptoms together with ketonuria, ketoacidosis, insulin autoantibody (IAA) positivity, or a low C-peptide level. Of children/adolescents who were under regular follow-up in 12 different centers in Turkey, those who were diagnosed with T1DM before January 1, 2007 and who had attended at least one follow-up visit were recruited. Patients with normal C-peptide levels, those who were considered to have maturity-onset diabetes of the young (MODY) based on the family history and clinical findings, those with T2DM, and those with a chronic disease (such as thalassemia, cystic fibrosis, drug-induced types) were excluded from the study. The data on the demographic and clinical characteristics of the patients, their anthropometric measurements, pubertal status, blood pressure values, insulin therapy regimens, the state of their metabolic control, lipid profile values, presence of concomitant autoimmune diseases, as well as acute and chronic complications were obtained from the patients’ files. The patients were grouped according to their hemoglobin A1c (HbA1c) levels in the past year. Those with good metabolic control (<7.5%) constituted Group 1, those with moderate metabolic control (between 7.6% and 9.0%) Group 2, and those with poor metabolic control (>9.0%) formed Group 3.

Standard deviation scores (SDS) for height, weight and body mass index (BMI) were assessed according to the Turkish population standards defined by Neyzi et al (2). Those with a BMI ≥95th percentile were considered obese (3). Pubertal status of each subject was determined using the Tanner criteria. Metabolic control and complications were defined according to the International Society for Pediatric and Adolescent Diabetes (ISPAD) consensus criteria (4,5).The biochemical criteria for the diagnosis of ketoacidosis were defined as hyperglycemia (blood glucose >11 mmol/L (>200 mg/dL), venous pH <7.3 or bicarbonate <15 mmol/L, ketonemia, and ketonuria (6).

Severe hypoglycemia was defined as the child having an altered mental status and being unable to cooperate, or his/her being semiconscious or unconscious, or in coma with or without convulsions and requiring parenteral therapy with glucagon or i.v. glucose (7).

The diagnosis of chronic lymphocytic thyroiditis and of Graves’ disease were based on physical examination, ultrasonography, and laboratory findings including the serum levels of free thyroxine (fT4), thyroid-stimulating hormone (TSH), anti-thyroperoxidase (anti-TPO), anti-thyroglobulin ?(anti-Tg), and TSH receptor antibodies (TRAb). The patients were assessed as euthyroid, or as having subclinical hypothyroidism, hypothyroidism, or hyperthyroidism according to their fT4 and TSH levels (8).

Gluten-sensitive enteropathy (GSE) was diagnosed by presence of IgA anti-tissue transglutaminase (IgA-tTG), anti-endomysium antibodies, and/or by biopsy findings (9).

Retinopathy was assessed using dilated fundoscopy, non-dilated fundoscopy, and fundus photography. Microaneurysm, hemorrhage, hard and soft exudates, intraretinal microvascular abnormalities, macular edema, proliferative retinopathy, and preproliferative retinopathy were accepted as the types of retinopathy (5).

Presence of proteinuria (≥500 mg/24 h) or albuminuria (≥300 mg/24 h) usually with hypertension was considered as evidence of diabetic nephropathy. Microalbuminuria was defined when one of the following was present: (i) an albumin excretion of 20-200 μg/min or 30-300 mg/day, (ii) an albumin/creatinine ratio of 2.5-25 mg/mmol or 30-300 mg/g in boys and 3.5-25 mg/mmol in girls in the morning urine sample. Persistent microalbuminuria was defined as albuminuria in at least two of three consecutive urine samples within 3-6 months (5).

Neuropathy was assessed by clinical examination and/or electromyography. Focal neuropathy (such as mononeuropathy/carpal tunnel syndrome), sensory-motor polyneuropathy, and autonomic neuropathy (such as postural hypotension, vomiting, bladder paresis, sweating) were accepted as the types of diabetic neuropathy (5).

Blood pressure measurements were performed after an adequate rest period and were repeated at least three times at 10-minute intervals. Subjects with age- and sex-adjusted systolic and/or diastolic blood pressures above the 95th percentile were considered hypertensive (10).

The presence of one or more of the following serum lipid values was considered as indicative of dyslipidemia: i) a morning fasting total cholesterol level higher than 5.18 mmol/L (200 mg/dL), ii) a low-density lipoprotein (LDL) level higher than 2.6 mmol/L (100 mg/dL), or iii) a high-density lipoprotein (HDL) level lower than 1.1 mmol/L (42 mg/dL) (5,11).

The authors confirmed in writing that they have complied with the World Medical Association Declaration of Helsinki regulations regarding ethical conduct of research involving human subjects and/or animals. The study was approved by the local ethics board and informed consent was obtained from the families of all patients.

## STATISTICAL ANALYSIS

Data analyses were performed with SPSS for Windows (version 15). Patient characteristics were determined using descriptive statistics. Independent t-test and one-way ANOVA test with Bonferroni’s correction for multiple comparisons were used to compare the means of groups when appropriate. A p-value of <0.05 was chosen to represent statistical significance, however, bivariate correlations were considered significant at p<0.017 (p<0.05/3=0.017). The values are expressed as mean±SD.

## RESULTS

The study included 1,032 patients (mean age, 12.5±4.1 years; 50.5% females, 49.5% males; 67.9% pubertal, 32.1% prepubertal), who were followed with a diagnosis of T1DM for a mean period of 4.7±3.2 years. The majority of the patients (70.4%) were aged between 10 and 20 years. The distribution of the patients according to age groups at the time of evaluation and at the time of diagnosis is presented in Table 1.

Obesity was present in 4.4% (n=38) of the patients. No statistically significant difference was found between the obese and non-obese T1DM patients in terms of age, diabetes duration and mean HbA1c levels of the past year (p>0.05) (Table 2).

Height SDS was below -2SD in 5% of the patients.Within the past one year, measurement of HbA1c as an indicator of metabolic control was performed three times in 35.5%, four times in 35.8%, twice in 18.4%, only once in 5.5% and more than four times in 4.8% the patients. According to these measurements, 31.6% the patients had good metabolic control, 33.6% had moderate metabolic control, and 34.8% had poor metabolic control. Mean HbA1c level was 8.5±1.8% at the last control visit and 8.4±1.6% in the past year. Mean age and mean duration of diabetes were shorter in Group 1 than in Groups 2 and 3 (Table 3). Moreover, it was found that the pubertal patients had significantly higher mean HbA1c levels in the past year as compared to the prepubertal patients (8.6±1.6 vs. 8.2±1.4, p=0.001).

Of the patients, 79.5% (694/873=67%) were on flexible insulin therapy (3 or more very fast-acting insulin analogues before meals + long-acting insulin), 5.9% were on insulin infusion (II) pump [continuous subcutaneous II (CSII)], and 3.2% were receiving multiple insulin therapy (NPH+ regular insulin before meals 3 times a day). Thirty (58.8%) of 51 patients using II pump had good metabolic control, whereas 12 (23.5%) had moderate metabolic control. Of the 694 patients receiving flexible insulin therapy, 206 (29.6%) had good metabolic control and 236 (34.0%) had moderate metabolic control. Insulin therapy regimens of the patients and their mean HbA1c levels in the past year are presented in Table 4.

**Concomitant Autoimmune Diseases**

Chronic lymphocytic thyroiditis was identified in 12% patients and Graves’ disease in 0.1%. While 954 (93.3%) of all patients were euthyroid, subclinical hypothyroidism was found in 29 (2.8%) patients, hypothyroidism in 37 (3.6%), and hyperthyroidism in 2 (0.2%) patients at the time of diagnosis or during follow-up.

Of the patients, 4.3% were under follow-up with a diagnosis of GSE. GSE was diagnosed by antibody positivity alone in 16.7% of the patients, by biopsy alone in 5.6%, and by both antibody positivity and biopsy in 77.8% of the patients.

**Acute Complications**

During the past year, severe hypoglycemia and ketoacidosis episodes were observed in 4.9% and 5.2% of the patients, respectively. No significant difference was found among the groups with good, moderate, and poor metabolic control in terms of the number of severe hypoglycemia episodes within the past year (p=0.58), while the number of ketoacidosis episodes was significantly lower in the group with good metabolic control as compared to groups with moderate and poor metabolic control (p=0.007 and p=0.008, respectively). There was no significant difference between the groups with moderate and poor metabolic control in terms of frequency of ketoacidosis (p=0.91; Table 5).

**Long-term Complications**

Mean total cholesterol, HDL- and LDL-cholesterol levels were 162.5±38.8 mg/dL, 57.1±14.4 mg/dL, and 88.2±28.8 mg/dL in the total group. These values were 166.76±39.21 mg/dL, 58.7±14.01 mg/dL, and 90.5±29.0 mg/dL in the boys; 158.14±37.9 mg/dL, 55.5±14.7 mg/dL, and 86.0±28.4 mg/dL in the girls. There was no significant difference between the genders. The rate of dyslipidemia was 17.5%, and 8.2% (n=14) of these patients were obese. The majority of patients with dyslipidemia (75.2%) were pubertal. Patients with dyslipidemia were found to be older compared to the cases without dyslipidemia (13.2±3.9 years vs. 12.4±4.1 years, p=0.02). No significant difference was determined between the patients with and without dyslipidemia in terms of diabetes duration (5.2±3.2 years and 4.7±3.2 years, respectively; p=0.91). However, there was a significant difference between the patients with and without dyslipidemia in terms of the mean HbA1c level in the past year (9.5±2.0% and 8.2±1.4%, respectively; p<0.001).

Focal neuropathy, sensory-motor neuropathy, and polyneuropathy were identified in 1.1%, 1.1%, and 0.5% of the patients, respectively. The patients with neuropathy were significantly older compared to those without (15.8±3.9 years and 12.4±4.1 years, respectively; p=0.001). Mean diabetes duration was significantly higher in patients with neuropathy than in those without (8.3±4.4 years and 4.6±3.1 years, respectively; p=0.00). There was no significant difference between these two groups in terms of mean HbA1c levels in the past year (8.5±1.4% in those with neuropathy and 8.4±1.6% in those without neuropathy; p=0.7).

Retinopathy was identified in 1.4% of the patients. Among them, 64.3% had microaneurysms, 14.3% hard and soft exudates, and 21.4% had proliferative retinopathy. Cataract was observed in 9.5% of the patients. The patients with retinopathy were significantly older than those without (16.7±3.9 years and 12.5±4.1 years, respectively; p=0.001). Mean diabetes duration of patients with retinopathy was also significantly longer than that of those without retinopathy (9.8±5.3 years and 4.7±3.2 years, respectively; p=0.00). There was no significant difference between these two groups regarding mean HbA1c levels in the past year (8.8±1.3% in those with retinopathy and 8.4±1.6% in those without retinopathy, p=0.3).

Diabetic nephropathy was not present in any of the patients, whereas persistent microalbuminuria was noted in 5.4%. The age of patients with and without microalbuminuria was 15.6±3.0 years and 12.4±4.1 years; the difference was significant (p=0.001). The duration of diabetes was significantly longer in patients with microalbuminuria compared to those without microalbuminuria (7.2±3.9 years and 4.6±3.1 years, respectively; p=0.001). No significant difference regarding glycemic control in the past year (HbA1c, 8.8±1.4% and 8.4±1.6%, respectively; p=0.05) was noted.

When the complications were evaluated according to the pubertal stages, nephropathy was present in 7.4% of pubertal patients and in 0.9% of prepubertal patients (p=0.001); retinopathy was present in 1.9% of pubertal patients and in 0.3% of prepubertal patients (p=0.07); neuropathy was present in 3.5% of pubertal cases and in 0.9% of prepubertal cases (p=0.02).

## DISCUSSION

In many developed countries, owing to adequate national registry systems, it has been possible to document the notable improvement achieved in recent years regarding the epidemiology, treatment, follow-up, and prevention of complications of T1DM. The EURODIAB study, which included most of the European countries and 30 million children, and the DIAMOND study, which included 84 million children from 112 centers in 57 countries, were initiated in the 1990s (12,13). Information about T1DM rapidly increased along with the data of these large, well-organized record systems. Developing a national registry system for T1DM, the incidence of which varies among races and countries, is of great importance for the prevention of microvascular and macrovascular complications (14).

The present study aimed to provide a first step in the development of a national T1DM registry system by evaluating the records of 1 032 T1DM patients who were diagnosed in childhood and followed at 12 centers in Turkey. Epidemiologic data of the patients, as well as their state of metabolic control, presence of concomitant other autoimmune diseases and that of microvascular and acute complications were assessed.

It is known that the incidence of T1DM increases with age and peaks in puberty. In the present study, 27.1% of the patients were in the 0-4.99 year age group, 42.6% were in the 5-9.99 year age group, and 27.9% were in the 10-14.99 year age group at the time of diagnosis. In the EURODIAB study, the distribution of patients among age groups at the time of diagnosis in 2005 was found as follows: 24% were in 0-4 year age group, 35% were in 5-9 year age group, and 41% were in 10-14 year age group. In that particular study, these rates were estimated to be 29%, 37% and 34%, respectively, in 2020 (15). In the DIAMOND study group, the incidence increased with age. The pooled data of the DIAMOND study group demonstrated that those in the 5-9 year age group had a 1.62 times higher risk [95% confidence intervals (CI), 1.57-1.66], and those in the 10-14 year age group had 1.94 times higher risk (95% CI, 1.89-1.98) than that of the 0-4 years olds (13). Similar to other studies showing no marked female dominance, the present study revealed a female-to-male ratio of 1.02 in frequency of autoimmune diseases, a finding which is on the contrary of female preponderance in other autoimmune diseases.

In the present study, mean HbA1c level was found to be significantly higher in the pubertal group as compared to the prepubertal group. This might have resulted from increased insulin resistance and impaired compliance with treatment in puberty. Approximately 80% of the patients in the present study were on flexible insulin therapy and 6% were on CSII. The rate of patients with good metabolic control was higher in the group receiving CSII as compared to the group receiving flexible insulin therapy. Previous studies have also demonstrated that CSII therapy is much more successful than flexible insulin therapy in achieving metabolic control and in preventing complications (16,17).

It is known that the prevalence rates of autoimmune thyroid disease and GSE are higher in patients with T1DM compared to the healthy population (18). In a study conducted in Turkey on 38 patients with T1DM, the rates of autoimmune thyroid disease and GSE were reported to be 31.5% and 7.8%, respectively (19). In another study from Turkey, Karaguzel et al (20) reported the rate of thyroid autoantibody positivity to be 38.6% and the rate of antiendomysial antibody positivity to be 3.5%. In their study, Mantovani et al (21) identified autoimmune thyroid disease in 16.7% of 474 patients with T1DM. In a multicenter study conducted in Germany, Kaspers et al (22) screened approximately 20 000 T1DM patients and reported the celiac antibody positivity to be 6.7%. In the present study, we demonstrated that 12% of the patients had autoimmune thyroid disease and 4.3% had GSE.

The rate of severe hypoglycemia was 4.9% in the present study group, and no significant difference was determined among the groups with good, moderate and poor metabolic control in terms of the number of hypoglycemic attacks in the past year (p>0.05). However, the frequency of hypoglycemia reported in this study might not reflect the real value, since it was calculated based on the data recorded by the patients or their relatives. Blasetti et al (23) performed a longitudinal study on 195 children with T1DM and followed them for 7.5 years. They reported that the rate of severe hypoglycemic attacks was 9.4% in one year and that hypoglycemic attacks were not associated with HbA1c levels, with daily insulin requirements, or with insulin regimens used. In a study performed by Rewers et al (24) on 1 243 T1DM patients aged 0 to 19 years, the frequency of severe hypoglycemia was reported to occur in 19% of the cases in the course of one year and was lower in females as compared to males. These authors showed that the frequency decreased with increasing age and increased with longer diabetes duration and with presence of concomitant psychiatric disease. They also did not find an association between hypoglycemia and HbA1c level or insulin dose. In the present study, the rate of ketoacidosis, another acute complication of T1DM, was found to be 5.2% in the course of one year and was significantly lower in the group with good metabolic control as compared to the groups with moderate or poor metabolic control. Rewers et al (24) reported this rate to be 8% in a year in both genders, and found that the risk increased with age in girls, as well as with high HbA1c levels and need for high insulin doses, length of diabetes duration, presence of psychiatric disease, and absence of health insurance. In a multicenter study based on the data of approximately 29 000 T1DM patients under the age of 20 years from Germany and Austria, total frequency of ketoacidosis was reported to be 5.9%; 4.9% of the ketoacidosis patients had presented with one episode, and 1.0% of the patients had presented with recurrent episodes (25). While the frequency of ketoacidosis was not found to be associated with the type of treatment and diabetes duration in the above-mentioned study, the rate of diabetic ketoacidosis was reported to be significantly higher in females, in children with migration background, and in those in their early teenage years (25).

T1DM is an important risk factor for dyslipidemia and cardiovascular diseases (5). In the present study, the prevalence of dyslipidemia was 17.5% and the majority (75.2%) of the cases with dyslipidemia were prepubertal. In their longitudinal study performed on 895 children and adolescents with T1DM, Marcovecchio et al (26) reported the rates of patients with high total cholesterol, high non-HDL cholesterol, high triglyceride, and high LDL-cholesterol levels to be 18.6%, 25.9%, 20.1%, and 9.6%, respectively. They also reported that sustained lipid abnormalities were associated with age, duration, BMI, and HbA1c. In the present study as well, dyslipidemia was found to be significantly associated with older age and higher HbA1c levels; however, no difference was found between those with and without dyslipidemia in terms of diabetes duration.

The frequency of diabetic neuropathy was found to be 2.7% in the present study. While neuropathy was associated with age and diabetes duration, no association was found with mean HbA1c level. In the 13th and 14th year of the Epidemiology of Diabetes Interventions and Complications (EDIC) study, the frequency of confirmed clinical neuropathy was reported to be 22% and 29% in the groups receiving intensive and conventional therapy, respectively (27). Lloyd et al (28) found the incidence of distal symmetric neuropathy to be 13% during a 4-year follow-up period. These studies demonstrated that neuropathy was associated with diabetes duration, age, and HbA1c level. The reason of lower neuropathy frequency in the present study might be the cross-sectional nature of the study and the young age of the patients.

The frequency of retinopathy, another microvascular complication of diabetes, was 1.4% in the present study group. In the 10th year of the EDIC study, the frequency of proliferative retinopathy was found to be 6.1% and 19.6%, and the frequency of non-proliferative retinopathy was found to be 6.3% and 19.6% in the groups receiving intensive and conventional therapy, respectively (29). Hammes et al (30) reported the frequency of any type of retinopathy to be 27.4% and the frequency of advanced retinopathy (severe non-proliferative or proliferative diabetic retinopathy) to be 8%. They demonstrated that the development of retinopathy was correlated with diabetes duration, HbA1c level, and smoking, and that the risk was higher for the male gender. In the present study, development of retinopathy was found to be associated with longer diabetes duration and older age, while no association was identified between development of retinopathy and mean HbA1c level. Again, the lower rate of retinopathy found in the present study might be attributed to the design of the study as well as to the younger age of the patients.

Persistent microalbuminuria was observed in 5.4% of the cases. Although the frequency of microalbuminuria was found to increase with age and was found to be more common in the pubertal cases, no association was present between microalbuminuria and mean HbA1c level. In their study on 27 805 patients with T1DM, Raile et al (31) determined the frequencies of microalbuminuria, macroalbuminuria, and end-stage renal insufficiency to be 3.3%, 0.28%, and 0.73%, respectively. In that particular study, they estimated the frequency of microalbuminuria to be 25.4% and macroalbuminuria or end stage renal insufficiency to be 9.4% at the end of a 40-year follow-up. They reported that diabetes duration, high blood pressure, and high LDL-cholesterol level were risk factors for microalbuminuria.

In conclusion, our findings indicate that although the majority of the patients in the present study were using insulin analogues, poor glycemic control was common, and chronic complications were encountered. These findings also show that to be able to provide appropriate therapy and care service for all T1DM patients, each country needs to develop its own registry system and a health care system enabling the monitoring of these patients for many years.

## Figures and Tables

**Table 1:**
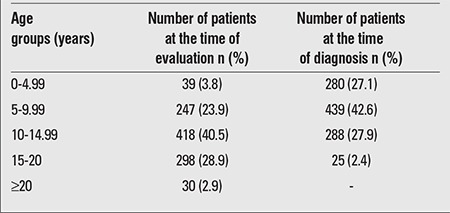
Distribution of the patients by age groups at the time ofevaluation and at the time of diagnosis

**Table 2:**
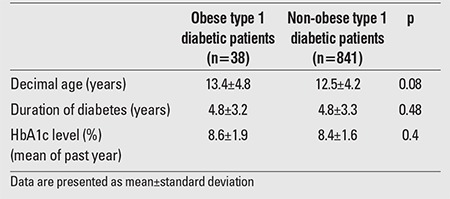
Age, duration of diabetes, and hemoglobin A1c (HbA1c)levels in obese and non-obese type 1 diabetic patients

**Table 3:**
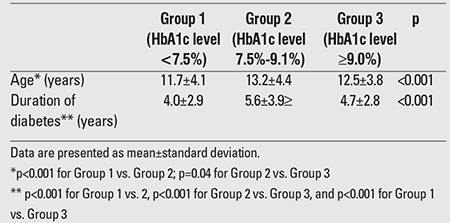
Age and diabetes duration of the patients according to theirmean hemoglobin A1c (HbA1c) levels

**Table 4:**
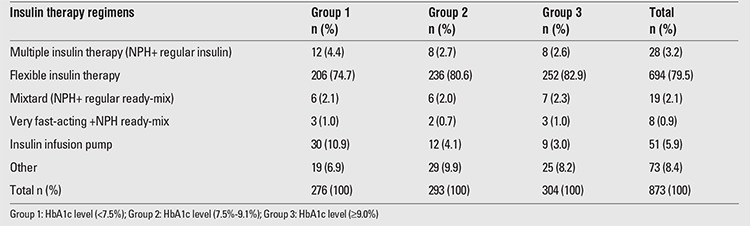
Insulin therapy regimens of the patients according to their mean hemoglobin A1c (HbA1c) levels in the past year

**Table 5:**
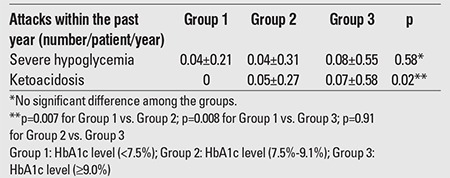
Mean numbers of severe hypoglycemia and ketoacidosis episodesaccording to glycemic control
